# Association between low-density lipoprotein cholesterol and the risk of bronchial asthma: a case–control study

**DOI:** 10.3389/fmed.2026.1808650

**Published:** 2026-08-03

**Authors:** Kexin Fan, Zhuoxiao Han, Tengfei Sun, Yingjie Zhang, Hua Qiao

**Affiliations:** 1 Department of Pulmonary and Critical Care Medicine, The First Hospital of Qinhuangdao, Qinhuangdao, Hebei, China; 2 Hebei Key Laboratory of Pathogenesis and Long-term Management of Lung Cancer and Chronic Airway Diseases, Qinhuangdao, Hebei, China; 3 Department of Gastroenterology, The First Hospital of Qinhuangdao, Qinhuangdao, Hebei, China

**Keywords:** bronchial asthma, LDL-C, non-linear relationship, PSM, RCS

## Abstract

This study investigated the association between low-density lipoprotein cholesterol (LDL-C) and the risk of bronchial asthma. A retrospective case–control study was conducted, including 944 patients with asthma and 1,580 controls. Subjects were divided by median LDL-C into high and low groups. Propensity score matching (PSM 1:1) was performed for age, gender, body mass index (BMI), family history, smoking, alcohol use, and diabetes, yielding 1,043 participants in each LDL-C group. Multivariate binary logistic regression was used post-PSM to assess the independent association between LDL-C and asthma risk. Stratified and interaction analyses were performed by age, gender, and BMI. Restricted cubic splines (RCS) with logistic regression were used to evaluate non-linear relationships and identify exploratory turning points. After PSM, multivariate analysis indicated LDL-C was independently associated with asthma risk (OR: 1.75, 95% CI: 1.47–2.09). Stratified analysis showed LDL-C was significantly associated with asthma risk in individuals aged <60 years (OR: 2.61, 95% CI: 2.04–3.36) but not in those ≥60 years (OR: 1.16, 95% CI: 0.91–1.49). Interaction analysis confirmed a significant difference between age strata (*p* < 0.05). No significant interactions were found for gender or BMI (*p* > 0.05). RCS analysis suggested a non-linear relationship, with an exploratory turning point at 3.62 mmol/L. This non-linearity was also present in both age subgroups (*p* < 0.05). Higher LDL-C was independently associated with asthma risk, and the estimated association curve was initially relatively stable with rising LDL-C and then became steeper once LDL-C exceeded 3.62 mmol/L.

## 1 Introduction

Bronchial asthma is a common chronic respiratory disease characterized by persistent airway inflammation, airflow limitation, and bronchial hyperresponsiveness. Globally, approximately 300 million people are affected by asthma, making it one of the most prevalent chronic inflammatory airway diseases (1). Rough estimates indicate that the prevalence of asthma among adults aged 20 and above in China is 4.26%. This represents a total of 45.7 million individuals, which imposes a substantial social and economic burden (2). Despite ongoing in-depth research into the pathogenesis of bronchial asthma, its precise etiology remains elusive. With the continuous improvement in living standards in China, significant changes in dietary habits have contributed to a rising number of individuals with dyslipidemia. Unfortunately, this population is at increased risk for conditions such as coronary heart disease and cancer.

The role of low-density lipoprotein cholesterol (LDL-C) in chronic diseases, such as coronary heart disease and cancer, has received considerable attention. LDL-C is widely recognized as a key factor in atherosclerosis, with elevated levels closely associated with an increased risk of cardiovascular diseases (3, 4). Additionally, the relationship between LDL-C and cancer extends beyond specific cancer types, involving potential roles in oncogenic mechanisms (5–8). Currently, the role of lipid metabolism, particularly LDL-C, in asthma is gaining focus. Several studies suggest a potential association between LDL-C and asthma development. A Mendelian randomization study by Liu et al. (9) also indicated a potential causal link between high LDL-C levels and an increased risk of asthma. Furthermore, studies in asthmatic children have found that LDL-C correlates with respiratory resistance and may serve as a reference indicator for asthma diagnosis and severity assessment (10). Moreover, an investigation by Wen et al. (11) concluded that among the U.S. asthmatic population, LDL-C levels exhibit a negative linear correlation with mortality. Thus, LDL-C is a metabolic marker that appears to play a role in the pathogenesis of various diseases. However, research on its role in asthma is still in its early stages of development.

Body fat distribution has been proven to be closely associated with adult asthma, and body mass index (BMI) alone may not fully capture metabolic heterogeneity relevant to asthma risk (12). LDL-C, although not a direct measure of adipose distribution, may partly reflect adverse metabolic milieu beyond BMI, which provides a new perspective for exploring the relationship between metabolic abnormalities and asthma.

This study investigates the association between metabolic dysregulation, particularly abnormal levels of LDL-C, and the risk of asthma. The objective is to provide a more scientific and precise theoretical foundation for the clinical prevention and management of asthma. The objective was to explore whether LDL-C is associated with asthma risk and to provide epidemiological evidence for future studies.

## 2 Materials and methods

### 2.1 Study participants

Patients hospitalized with bronchial asthma at The First Hospital of Qinhuangdao between January 2018 and January 2024 were selected as the case group. Individuals confirmed to be free of asthma through health examinations during the same period were selected as the control group.

## 3 Inclusion and exclusion criteria

### 3.1 Case group

Inclusion criteria: (1) age ≥ 18 years; (2) Diagnosis conforming to GINA 2024 diagnostic criteria for bronchial asthma (13), confirmed by typical variable respiratory symptoms and objective examinations such as bronchial dilation test.

Exclusion criteria: (1) critically ill condition requiring ICU admission; (2) Requirement for mechanical ventilatory support; (3) Comorbid malignant tumor(s); (4) Use of LDL-C-lowering drugs; (5) Incomplete clinical data.

### 3.2 Control group

Inclusion criteria: (1) age ≥ 18 years; (2) Confirmed absence of bronchial asthma via health examination at our hospital during the same period.

Exclusion criteria: (1) comorbid malignant tumor(s); (2) Use of LDL-C-lowering drugs; (3) Incomplete data.

This study was approved by the Ethics Committee of The First Hospital of Qinhuangdao (Ethics Approval Number: 2024 K016).

### 3.3 Laboratory testing

Blood samples were collected at 8:00 a.m. after an 8-h overnight fast when asthma patients were in stable condition, and the serum levels of LDL-C, high-density lipoprotein cholesterol (HDL-C), and uric acid (UA) were measured.

### 3.4 Statistical analysis

Continuous variables were described as mean ± standard deviation if normally distributed, or as median (interquartile range) if non-normally distributed. For comparisons across multiple groups, one-way ANOVA was used for normally distributed data, with the LSD test applied for homogeneous variances and Tamhane’s T2 test for heterogeneous variances. The Kruskal-Wallis test was employed for non-normally distributed data. Categorical variables were summarized as counts (percentages) and compared using the R × C chi-square test.

To assess the impact of LDL-C levels on asthma risk, we first divided LDL-C into quartiles to examine baseline characteristics across different LDL-C groups, highlighting distribution differences. Propensity score matching (PSM) was then applied with a 1:1 matching ratio and a caliper of 0.02 to control for potential confounders, including age, gender, BMI, family history of asthma, smoking, alcohol consumption, and diabetes. After matching, all standardized mean differences (SMDs) were below 0.1, indicating excellent balance. After successful matching, logistic regression was performed to explore the association between LDL-C and asthma risk, a common method to minimize confounding bias in observational studies.

Subsequent stratified analyses and interaction tests based on age, gender, and BMI were conducted on the PSM-matched sample to investigate whether the LDL-C-asthma association varied across subgroups. Additionally, a restricted cubic splines (RCS) analysis was performed using continuous LDL-C data (not the matched sample) to model the potential non-linear relationship between LDL-C and asthma risk. Four knots were selected at the 5th, 35th, 65th, and 95th percentiles. We further explored threshold effects by testing LDL-C (from the 10th to the 90th) as potential exploratory inflection points, comparing piecewise linear models with the standard linear model via a log-likelihood ratio test.

All analyses were conducted using R version 4.2.1. A two-sided *p*-value < 0.05 was considered statistically significant.

## 4 Results

### 4.1 Comparison of baseline characteristics

A total of 1,016 patients with asthma were initially enrolled as the case group, and 1,609 individuals were included as the control group. Subsequently, in the case group, 39 participants using LDL-C–lowering medications, 3 participants with malignant tumors, and 30 participants with incomplete data were excluded. In the control group, 8 participants using LDL-C–lowering medications, 1 participant with a malignant tumor, and 20 participants with incomplete data were excluded.

A total of 944 eligible patients with bronchial asthma were included in the case group, along with 1,580 individuals without bronchial asthma confirmed during the same period, forming the control group. Based on LDL-C quartiles, the study population was categorized into four groups (Q1, Q2, Q3, and Q4). A comparative analysis of baseline characteristics revealed statistically significant differences (*p* < 0.05) in age, gender, BMI, diabetes status, smoking status, alcohol consumption, and UA levels among the groups. No significant differences were observed for the other variables (*p* > 0.05), as detailed in Table 1.

**Table 1:** General characteristics of different LDL-C groups.

Variable	Q1	Q2	Q3	Q4	*p*
*n*	640	629	626	629	
Age, ≥60 years (*n*, %)	233 (36.4)	151 (24.0)	177 (28.3)	314 (49.9)	<0.001
Gender, males (*n*, %)	325 (50.8)	353 (56.1)	365 (58.3)	317 (50.4)	0.007
BMI (*n*, %)					0.030
<18.5 kg/m^2^	33 (5.2)	23 (3.7)	6 (1.0)	21 (3.3)	
≥18.5 kg/m^2^ to < 25 kg/m^2^	331 (51.7)	299 (47.5)	291 (46.5)	299 (47.5)	
≥25 kg/m^2^ to < 30 kg/m^2^	220 (34.4)	247 (39.3)	257 (41.1)	240 (38.2)	
≥30 kg/m^2^	56 (8.8)	60 (9.5)	72 (11.5)	75 (11.9)	
Diabetes (*n*, %)	92 (14.4)	51 (8.1)	43 (6.9)	71 (11.3)	<0.001
Smoking (*n*, %)	93 (14.5)	87 (13.8)	108 (17.3)	127 (20.2)	0.014
Drinking (*n*, %)	40 (6.3)	45 (7.2)	44 (7.0)	73 (11.6)	0.005
Family history (*n*, %)	8 (1.3)	15 (2.4)	14 (2.2)	21 (3.3)	0.172
HDL-C (mmol/L)	1.21 ± 0.36	1.25 ± 0.32	1.25 ± 0.29	1.25 ± 0.31	0.161
Uric acid (μmol/L)	322.93 ± 96.18	339.72 ± 97.77	345.67 ± 96.50	346.59 ± 87.34	<0.001

BMI, body mass index; LDL-C, low-density lipoprotein cholesterol; HDL-C, high-density lipoprotein cholesterol.

### 4.2 Baseline characteristics before and after PSM

Study participants were divided into two groups, high and low LDL-C, based on the median LDL-C level. Subsequently, 1:1 propensity score matching was performed for age, gender, BMI, family history of asthma, smoking history, alcohol consumption history, and diabetes. A total of 1,043 subjects were successfully matched in both the high LDL-C group and the low LDL-C group.

Before PSM, significant differences (*p* < 0.05) were observed between the two groups in age, BMI, family history, smoking history, alcohol consumption, and diabetes. After matching, these baseline characteristics showed no statistically significant differences (*p* > 0.05). After matching, all SMDs were below 0.1, indicating excellent balance. Detailed results are presented in Table 2.

**Table 2:** General characteristics between the high-LDL-C group and low-LDL-C group.

Variables	Pre-PSM		SMD	*p*	After PSM		SMD	*p*
Subgroup	High-LDL-C group *n* = 1,262	Low-LDL-C group *n* = 1,262			High-LDL-C group *n* = 1,043	Low-LDL-C group *n* = 1,043		
Age ≥60 (*n*, %)	491 (38.9%)	383 (30.3%)	0.181	<0.001	341 (32.7%)	335 (32.1%)	0.012	0.815
Gender, males (*n*, %)	686 (54.4%)	673 (53.3%)	0.021	0.632	601 (57.6%)	566 (54.3%)	0.068	0.134
BMI (*n*, %)			0.151	0.002			0.089	0.246
<18.5 kg/m^2^	27 (2.1%)	54 (4.3%)			21 (2.0%)	19 (1.8%)		
≥18.5 kg/m^2^ to < 25 kg/m^2^	595 (47.1%)	630 (49.9%)			513 (49.2%)	508 (48.7%)		
≥25 kg/m^2^ to < 30 kg/m^2^	496 (39.3%)	463 (36.7%)			428 (41.0%)	409 (39.2%)		
≥30 kg/m^2^	144 (11.4%)	115 (9.1%)			81 (7.8%)	107 (10.3%)		
Diabetes (*n*, %)	112 (8.9%)	144 (11.4%)	0.084	0.041	91 (8.7%)	86 (8.2%)	0.017	0.694
Smoking (*n*, %)	234 (18.5%)	179 (14.2%)	0.118	0.004	176 (16.9%)	144 (13.8%)	0.085	0.052
Drinking (*n*, %)	116 (9.2%)	85 (6.7%)	0.091	0.027	86 (8.2%)	69 (6.6%)	0.062	0.156
Family history (*n*, %)	39 (3.1%)	22 (1.7%)	0.088	0.038	10 (1.0%)	15 (1.4%)	0.044	0.314

BMI, body mass index; LDL-C, low-density lipoprotein cholesterol; PSM, propensity score matching; SMD, standardized mean difference.

### 4.3 Analysis of the linear relationship between LDL-C and asthma risk after PSM

Following PSM, logistic regression analyses were performed to evaluate the association between LDL-C and asthma risk. In Model 1 (unadjusted), LDL-C was significantly associated with asthma risk (OR 2.01, 95% CI, 1.78–2.27). In Model 2, adjusted for age, gender, BMI, family history of asthma, smoking history, alcohol consumption, and diabetes, LDL-C remained significantly associated with asthma risk (OR 2.35, 95% CI, 2.02–2.73). In Model 3, which was further adjusted for HDL-C and UA, LDL-C persisted as independently associated with asthma risk (OR 1.75, 95% CI 1.47–2.09). Detailed results are presented in Table 3.

**Table 3:** Analysis of the linear relationship between LDL-C and asthma risk.

Variables	Model 1	*p*	Model 2	*p*	Model 3	*p*
	OR (95%CI)		OR (95%CI)		OR (95%CI)	
LDL-C	2.01 (1.78, 2.27)	<0.001	2.35 (2.02, 2.73)	<0.001	1.75 (1.47, 2.09)	<0.001

Model 1: unadjusted.

Model 2: adjusted for age, gender, BMI, family history of asthma, smoking, alcohol consumption, and diabetes.

Model 3: further adjusted for HDL-C and uric acid based on Model 2.

LDL-C, low-density lipoprotein cholesterol; OR, odds ratio; CI, confidence interval.

### 4.4 Stratified and interaction analyses

**Figure 1:**
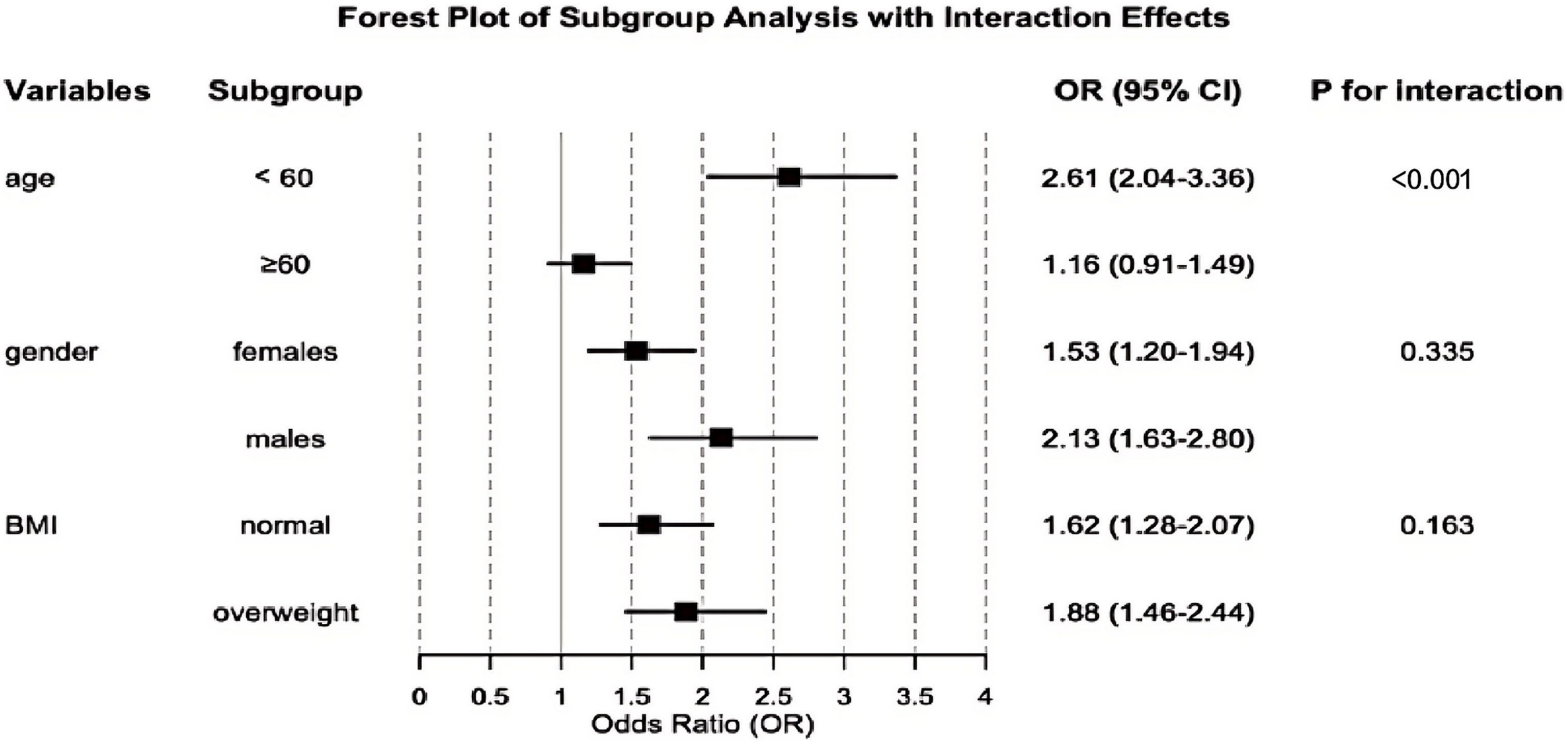
Forest plot of subgroup analysis with interaction effects. BMI, body mass index; OR, odds ratio; CI, confidence interval.

Stratified analyses assessed the association between LDL-C and asthma risk across subgroups defined by age, gender, and BMI, followed by formal tests for interaction. The results revealed a significant association between LDL-C and asthma risk in individuals aged <60 years (OR 2.61, 95% CI 2.04–3.36). In contrast, no significant association was observed in individuals aged 60 years or older (OR, 1.16, 95% CI, 0.91–1.49). This effect modification by age was statistically significant, as confirmed by the interaction test (*p* < 0.05). LDL-C remained significantly associated with asthma across all subgroups of gender and BMI. However, no statistically significant interaction effects were found for either gender or BMI (*p* > 0.05 for both). These results are summarized in Figure 1.

### 4.5 Non-linear relationship and threshold effect

**Figure 2:**
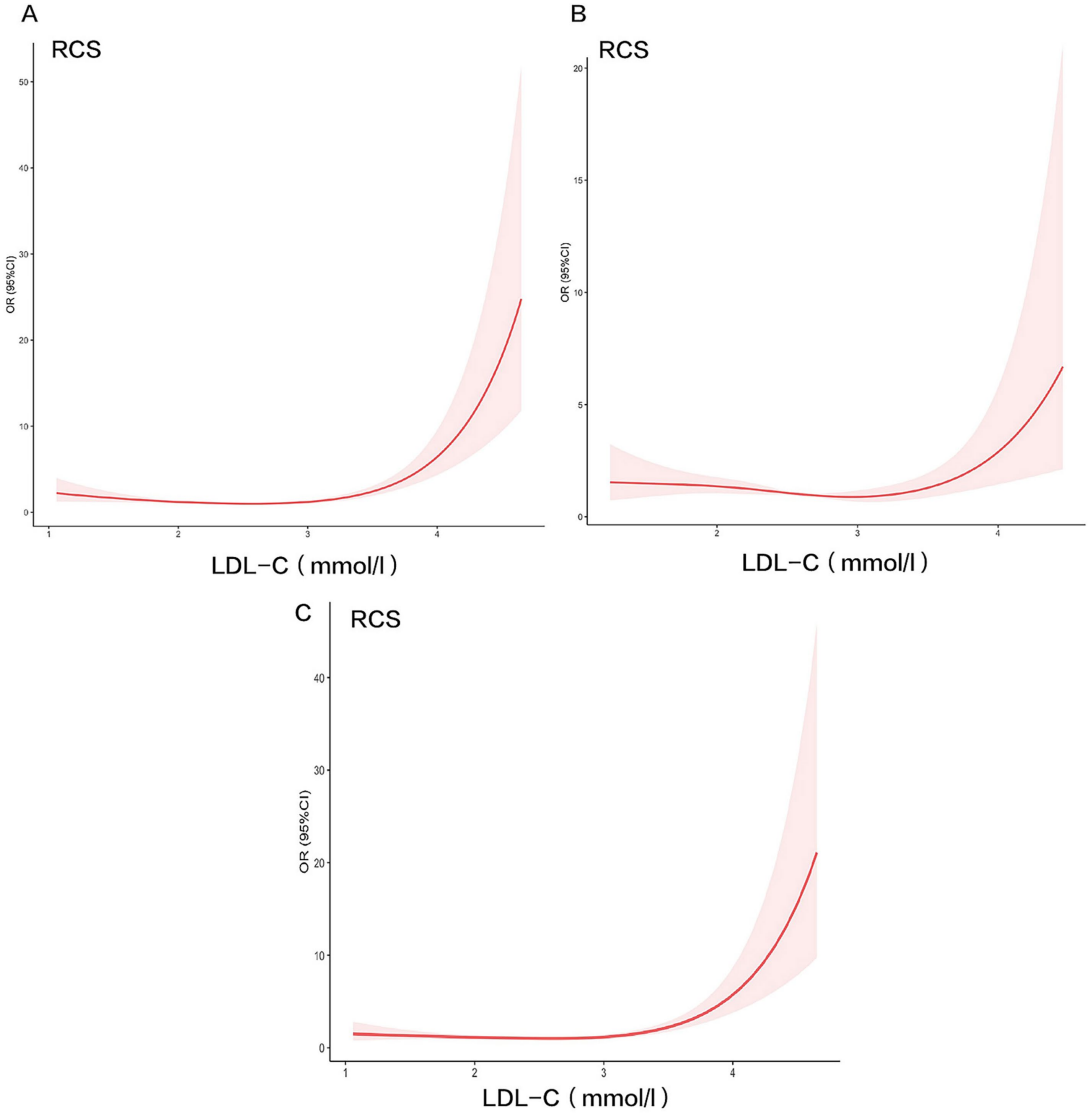
Non-linear relationship between LDL-C and asthma risk. **(A)** < 60 years; **(B)** ≥ 60 years; **(C)** Overall population. The restricted cubic spline model in the overall population was adjusted for age, gender, body mass index, diabetes, family history, smoking, drinking, high-density lipoprotein cholesterol, and uric acid. In age-stratified analyses, models were adjusted for all covariates except age. Solid lines represent OR curves; shaded regions indicate 95% confidence intervals. LDL-C, low-density lipoprotein cholesterol; RCS, restricted cubic splines; OR, odds ratio; CI, confidence interval.

RCS analysis, combined with logistic regression, revealed a significant non-linear association between LDL-C and asthma risk after adjusting for confounders, including age, gender, BMI, diabetes, family history of asthma, smoking, drinking, HDL-C and UA (*p* < 0.05). As LDL-C levels increased, the estimated association curve initially remained relatively stable and subsequently became steeper (Figure 2).

Given the significant interaction effect between LDL-C and age identified in previous stratified analysis (*p* < 0.05)—particularly the absence of a substantial association in individuals aged ≥60 years (OR 1.16, 95% CI 0.91–1.49)—we further performed age-stratified RCS analyses. Notably, these analyses confirmed a non-linear relationship between LDL-C and asthma risk in both age subgroups (<60 and ≥60 years, *p* < 0.05 for both), as detailed in Figure 2.

The nonlinear analysis revealed a distinct pattern in the participants aged ≥60 years, the linear term was not statistically significant, whereas the spline model suggested a possible nonlinear association. Therefore, the relationship in this subgroup may be more complex than a simple linear pattern.

### 4.6 Threshold effect analysis

In the overall population, the estimated association between LDL-C and asthma risk showed a gradual increase below the exploratory turning point of 3.62 mmol/L and appeared steeper once LDL-C levels reached or exceeded this value. In the subgroup aged <60 years, a potential threshold effect was suggested in the relationship between LDL-C levels and asthma risk; the exploratory turning point was 3.61 mmol/L. Among participants aged 60 years or older, a potential threshold effect was suggested at a concentration of 3.60 mmol/L. Below this value, the estimated association curve increased slowly with rising LDL-C levels, whereas beyond it, the curve became steeper. However, the wide confidence intervals above the turning point suggest that the threshold results should be interpreted as exploratory and potentially unstable rather than definitive evidence of a biological cutoff. The detailed results of these threshold analyses are presented in Table 4.

**Table 4:** Threshold effect analysis of LDL-C and asthma risk in different age groups.

LDL-C, mmol/L	Total (*n* = 2086)	*p*	< 60 (*n* = 1,410)	*p*	≥60 (*n* = 676)	*p*
	OR (95%CI)		OR (95%CI)		OR (95%CI)	
Turning point	3.62		3.61		3.60	
< Turning point	1.34 (1.08–1.65)	< 0.001	0.84 (0.62–1.15)	0.280	1.06 (1.02–1.15)	0.002
> Turning point	12.88 (5.11–34.01)	< 0.001	10.69 (2.51–65.10)	0.004	10.49 (2.51–62.14)	0.004
*p*		< 0.001		0.001		< 0.001

LDL-C, low-density lipoprotein cholesterol; OR, odds ratio; CI, confidence interval.

### 4.7 Sensitivity analysis

A sensitivity analysis was performed after further excluding 52 participants with documented oral corticosteroid use within the previous 6 months. After PSM, baseline characteristics were well balanced (Supplementary Table S1). Logistic regression confirmed that high LDL-C remained significantly associated with asthma risk (Supplementary Table S2). RCS analysis showed a similar non-linear pattern between LDL-C and asthma risk, as detailed in Supplementary Figure S1. These results support the robustness of the main findings.

## 5 Discussion

This study demonstrates that LDL-C is independently associated with asthma risk after adjusting for confounders, which is consistent with existing literature. For instance, an analysis by Peng J et al. reported higher LDL-C levels in asthmatic patients compared to non-asthmatic controls (14). Elevated cholesterol levels stimulate pro-inflammatory processes and enhance the secretion of related cytokines. A nationwide Danish study of 29,851 children treated with inhaled corticosteroids further substantiates this relationship, showing that elevated LDL-C was associated with a 2.2-fold increased odds of severe asthma (15). Interestingly, a Mendelian randomization study simulating statin-like inhibition of HMG-CoA reductase (HMGCR) found that genetic inhibition had no significant effect on most immune-related diseases. However, it was positively associated with asthma risk in East Asian populations (16). This suggests that genetic proxies related to LDL-C lowering via HMGCR were associated with asthma risk. Further genetic evidence comes from studies on PCSK9 variants, where specific mutations associated with lower LDL-C levels were linked to an increased risk of asthma (17). This underscores the potential role of genetic regulation of LDL-C in respiratory diseases.

LDL-C also appears to correlate with asthma severity. In a study of 167 adult asthmatics, LDL-C levels showed a significant association with fractional exhaled nitric oxide, indicating a link between cholesterol metrics and airway inflammation (18). Another study involving 159 atopic asthma patients found a negative correlation between serum LDL-C and forced expiratory volume in 1 s, suggesting that higher LDL-C may reflect limited airflow (19). Lifestyle interventions, such as increased vitamin intake, reduced LDL-C levels, and limited consumption of oxidized lipids, are beneficial for lowering asthma risk and symptom severity (20). Collectively, these studies support our conclusion and indicate the potential clinical relevance of LDL-C in asthma diagnosis and management.

Potential biological links between LDL-C and asthma may be considered from the following perspectives. First, LDL-C may be linked to the inflammatory response in asthma. Studies have shown that oxidized low-density lipoprotein (ox-LDL) is associated with declined lung function, enhanced inflammation, and oxidative stress in chronic obstructive pulmonary disease (21). Similar mechanisms may also operate in asthma, as ox-LDL can aggravate airway inflammation by promoting the release of pro-inflammatory cytokines. Second, elevated LDL-C levels may be related to the proliferation of airway smooth muscle cells and angiogenesis, both of which are key features of asthmatic airway remodeling (22). Such structural changes may be related to airway narrowing and airflow limitation, thereby potentially worsening asthma symptoms. Third, a Mendelian randomization study suggested that LDL-C may act as a mediator in the causal pathway between systemic lupus erythematosus and asthma, accounting for approximately 6.15% of the total effect (23). This finding implies that LDL-C may be associated with cardiovascular diseases and may also be involved in the pathophysiology of asthma.

Distinct from previous studies, our research employed PSM, stratified analyses, and interaction tests, yielding more refined insights. We identified an age-specific relationship: LDL-C was independently associated with asthma risk in individuals under 60 years, but not in those aged 60 or older, with a statistically significant interaction effect. For participants aged 60 years or older, the estimated asthma-risk curve increased gradually with LDL-C levels below 3.60 mmol/L and appeared steeper beyond this point. In the presence of a non-linear relationship and a potential threshold effect, a single linear model may underestimate or mask the association strength in some intervals, so the non-significant linear model results in the elderly group do not mean that there is no LDL-C-asthma association in this group; however, this non-linear finding should still be regarded as an exploratory signal rather than a definite conclusion. Notably, an LDL-C level of 3.60 mmol/L approaches the established cardiovascular risk threshold of 3.40 mmol/L (24), suggesting potential shared physiological mechanisms across conditions. The threshold value of approximately 3.60 mmol/L is close to values reported in some cardiovascular studies, although it should not be interpreted as an established clinical cutoff.

However, some studies have reported conflicting findings. For instance, an analysis of the NHANES database involving 11,662 children and 12,179 adolescents found no significant association between elevated LDL-C and current asthma after adjusting for gender, race, and other factors (25). These discrepancies may stem from methodological variations, differences in population characteristics (e.g., age, ethnicity, geographic region), and the control for confounding factors. Moreover, asthma is a complex and heterogeneous disease involving multiple pathogenic pathways. Thus, LDL-C may represent just one component of this intricate network, potentially interacting with other factors and adding complexity to studying its specific role. Furthermore, the precise molecular mechanisms through which LDL-C influences asthma remain incompletely understood, warranting further investigation to clarify the exact relationship. Our study possesses several strengths that enhance the robustness of the findings: (1) Rigorous stratified and interaction analyses were conducted to explore effect modification. (2) Propensity score matching based on age, gender, and BMI was performed to minimize confounding. (3) Non-linear and threshold effect analyses were employed, providing a more comprehensive examination of the association between LDL-C and asthma.

Nevertheless, several limitations should be acknowledged. First, given the retrospective case–control design, a causal relationship between LDL-C and asthma cannot be established. Second, because asthma itself and its treatments, particularly glucocorticoids, may affect lipid metabolism, reverse causality cannot be excluded. Third, the selection of asthma patients as cases and individuals undergoing routine health examinations as controls may have introduced selection bias because cases and controls came from different source populations. Although PSM was applied and sensitivity analyses were conducted, residual confounding and systematic differences in health behaviors, comorbidity profiles, and healthcare utilization may still remain and could not be fully eliminated. In addition, data on diet, physical activity, and socioeconomic status were not available, which may have further contributed to unmeasured confounding. Therefore, prospective studies with more comprehensive covariate assessment, as well as mechanistic investigations, are warranted to confirm our findings and clarify the underlying mechanisms.

## 6 Conclusion

In this study, higher LDL-C was independently associated with asthma risk, and a non-linear relationship was observed. Further prospective cohort and mechanistic studies are warranted to validate these findings.

## Data Availability

The original contributions presented in the study are included in the article/Supplementary material, further inquiries can be directed to the corresponding author.
