# Supplementary material for: Association between low-density lipoprotein cholesterol and the risk of bronchial asthma: a case–control study
Source: Front Med (Lausanne). 2026 Aug 3;13:1808650. doi: 10.3389/fmed.2026.1808650 (PMC13478071; doi:10.3389/fmed.2026.1808650)
Supplement: Supplementary file 1 [file Table_1.docx]

**Table S1.** Sensitivity analysis of general characteristics between the high-LDL-C group and low-LDL-C group.

| Variables | pre-PSM |  | SMD | *P* | after PSM |  | SMD | *P* |
| --- | --- | --- | --- | --- | --- | --- | --- | --- |
| Subgroup | High-LDL-C group  n=1236 | Low-LDL-C group  n=1236 |  |  | High-LDL-C group  n=1032 | Low-LDL-C group  n=1032 |  |  |
| Age ≥60 (n, %) | 479 (38.5%) | 379 (30.5%) | 0.170 | <0.001 | 314 (30.4%) | 326 (31.6%) | 0.066 | 0.885 |
| Gender, males (n, %) | 678 (54.5%) | 662 (53.2%) | 0.026 | 0.520 | 579 (56.1%) | 576 (55.8%) | 0.008 | 0.627 |
| BMI (n, %)  <18.5 kg/m^2^  ≥18.5 kg/m^2^<25 kg/m^2^  ≥25 kg/m^2^<30 kg/m^2^  ≥30 kg/m^2^ | 26 (2.1%)  586(47.1%)  493(39.6%)  139 (11.2%) | 54 (4.3%) 618(49.7%) 457 (36.7%) 115 (9.2%) | 0.152 | 0.003 | 25 (2.4%)  494 (47.9%)  401 (38.9%)  112 (10.9%) | 26 (2.5%)  502 (48.6%)  393 (38.1%)  111 (10.8%) | 0.079 | 0.911 |
| Diabetes (n, %) | 112 (9.0%) | 142 (11.4%) | 0.080 | 0.047 | 91 (8.8%) | 92 (8.9%) | 0.001 | 0.999 |
| Smoking (n, %) | 224 (18.0%) | 177 (14.2%) | 0.103 | 0.010 | 163 (15.8%) | 154 (14.9%) | 0.024 | 0.999 |
| Drinking (n, %) | 107 (8.6%) | 84 (6.8%) | 0.069 | 0.083 | 74 (7.2%) | 72 (7.3%) | 0.017 | 0.513 |
| Family history  (n, %) | 38 (3.1%) | 22 (1.8%) | 0.084 | 0.035 | 12 (1.2%) | 21 (2.0%) | 0.036 | 0.706 |

BMI, body mass index; LDL-C, low-density lipoprotein cholesterol; PSM, propensity score matching; SMD, standardized mean differences.
