# Supplementary material for: Association between low-density lipoprotein cholesterol and the risk of bronchial asthma: a case–control study
Source: Front Med (Lausanne). 2026 Aug 3;13:1808650. doi: 10.3389/fmed.2026.1808650 (PMC13478071; doi:10.3389/fmed.2026.1808650)
Supplement: Supplementary file 2 [file Table_2.docx]

**Table S2.** Sensitivity analysis of association between LDL-C and asthma risk after PSM

|  | Model 1 |  | Model 2 |  | Model 3 |  |
| --- | --- | --- | --- | --- | --- | --- |
|  | OR (95%CI) | *P* | OR (95%CI) | *P* | OR (95%CI) | *P* |
| LDL-C | 1.64 (1.36, 1.98) | <0.001 | 2.03 (1.62, 2.54) | <0.001 | 1.53 (1.19, 1.97) | 0.001 |

Model 1: unadjusted.

Model 2: adjusted for age, gender, BMI, family history of asthma, smoking, alcohol consumption, and diabetes.

Model 3: further adjusted for HDL-C and uric acid based on Model 2.

LDL-C, low-density lipoprotein cholesterol; PSM, propensity score matching; OR, odds ratio; CI, confidence interval.
